# Dual-Mode Detection of Perfluorooctanoic Acid Using Up-Conversion Fluorescent Silicon Quantum Dots–Molecularly Imprinted Polymers and Smartphone Sensing

**DOI:** 10.3390/foods15020331

**Published:** 2026-01-16

**Authors:** Hongli Ye, Xinran Wang, Xiangqian Xu, Hongyang Xu, Rui Yuan, Ping Cheng

**Affiliations:** 1 Laboratory of Aquatic Product Quality, Safety and Processing, Key Laboratory of Oceanic and Polar Fisheries, Ministry of Agriculture and Rural Affairs, East China Sea Fisheries Research Institute, Chinese Academy of Fishery Sciences, Shanghai 200090, China; 2 College of Food Science and Engineering, Dalian Ocean University, Dalian 116023, China; 3 School of Materials and Chemistry, University of Shanghai for Science and Technology, Shanghai 200093, China

**Keywords:** rapid detection, up-conversion, silicon quantum dots, molecular imprinting technology, perfluorooctanoic acid, smartphone sensing

## Abstract

Perfluorooctanoic acid (PFOA) is a persistent and bioaccumulative hazardous pollutant, presenting substantial threats to the environment and human health. The dual-mode, portable, sensitive, low-background, and cost-effective detection methods for PFOA were developed by integrating up-conversion fluorescent silicon quantum dot–molecularly imprinted polymer (MIPs) with a smartphone-based sensing system. The interaction between PFOA and MIPs resulted in a fluorescence quenching with a range of 2–20 µmol/L and a limit of detection (LOD) of 37.5 nmol/L for the low-background up-conversion fluorescence detection of PFOA, whereas the portable smartphone sensing platform enabled the detection of PFOA with a linear range of 0–5 µmol/L and a LOD of 73.9 nmol/L. Furthermore, the established methods were successfully applied to the detection of PFOA in environmental waters and food samples. This study provides the dual-mode, portable, novel, practical and low-background approaches for the detection of PFOA in the environment and food products.

## 1. Introduction

As a group of highly fluorinated organic compounds, polyfluoroalkyl substances (PFASs) have been widely used in industrial production, including pesticides, leather, food packaging, as well as in people’s daily lives for their remarkable amphiphilicity, chemical stability, thermal resistance, and surface activity [1,2,3]. However, owing to the strong C-F bond, PFASs exhibit high stability and persistence in the environment, which have drawn significant attention because of their difficult degradation, long-distance migration, wide existence, and even potential threat to human health and ecological systems [4,5,6]. Chronic exposure to certain PFAS congeners has been causally associated with an increased risk of carcinogenesis, endocrine disruption, altered physiological homeostasis, immunosuppression, and heightened susceptibility to infectious and chronic diseases [7,8]. Perfluorooctanoic acid (PFOA) is one of the highly concentrated PFASs and is easily found in the environment and food chains because of its anionic and amphiphilic properties [9,10]. Human exposure occurs mainly through drinking water and food consumption, leading to bioaccumulation in the vital organs such as the brain, kidneys, liver and lungs, thereby posing serious health risks [11,12]. Hence, it is crucial to develop in situ analytical methods for real-time monitoring of the PFOA level in environmental and food samples.

A serial of techniques is widely used to analyze PFOA for high sensitivity, including high-performance liquid chromatography (HPLC) [13], gas chromatography–mass spectrometry (GC-MS) [14], and high-performance liquid chromatography tandem mass spectrometer (HPLC-MS/MS) [15,16], yet limits its further on-site application by time-consuming procedures, high cost, large-scale equipment, professional technical personnel, and dependent laboratory [17]. The complexity of sample matrices and the diversity of interfering components further exacerbated the challenge, highlighting the urgent demand for advanced technologies capable of analyzing trace and even ultra-trace levels of PFOA in environmental water bodies and foods. Fluorescence sensing based on nanoprobes is characterized by low cost, simple preparation, rapid response, lossless analysis, in situ testing, and high sensitivity, and has been applied in numerous fields [18,19]. However, challenges persist, encompassing background interference within intricate matrices and the deficiency of portability. As a promising fluorescence probe, silicon quantum dots (SiQDs) have received increasing attention because of featuring the outstanding metrics of abundant sources, simple preparation, tunable surface properties, superior optical characteristics, strong photobleaching resistance, and low biotoxicity [20,21,22]. Moreover, SiQDs exhibit the excellent up-conversion property, which might be attributed to the two-photon absorption process [23,24]. Up-conversion-based fluorescence sensing could minimize autofluorescence interference from background signals and enhance tissue permeability depth in complex matrices to improve the sensitivity [25,26]. In recent years, smartphone-based biosensing technology has demonstrated great potential as an alternative to conventional laboratory methods, with remarkable progress in point-of-care diagnosis, environmental monitoring, and food analysis [27,28]. These smartphone-based approaches generally simplify detection workflows and offer user-friendly operation. The combination of smartphones with highly sensitive and selective chemical sensors is expected to provide powerful tools for on-site assessment of environmental water quality and food safety.

Herein, we proposed the dual-mode approach for PFOA detection in environmental and food samples based on silicon quantum dots molecularly imprinted polymers (MIPs) and their low-background up-conversion fluorescence, together with the portability of smartphones, thereby establishing a robust detection system that synergistically combines high sensitivity, exceptional selectivity, portability, and cost-effectiveness. Significantly, the prepared MIPs demonstrated abundant surface functional groups, satisfactory selectivity and anti-interference capabilities. Up-conversion fluorescence facilitates the transformation of low-energy near-infrared light into high-energy visible light. This characteristic intrinsically eliminates the interference of background fluorescence originating from biomacromolecules or natural fluorophores in environmental water and food matrices. Optical spectra and fluorescence decay curves indicated that electron transfer accounted for the fluorescence quenching of MIPs in the presence of PFOA. The alteration of fluorescence intensity of MIPs exhibited a good linear relationship with the varied concentration of PFOA in the range of 2.0–20 μM. Furthermore, with the assistance of a smartphone, a portable sensing method was also established. By utilizing the smartphone camera to replace the large-scale instruments and equipment needed for traditional fluorescence detection, the miniaturization and on-site portability of the detection system are achieved. And exploitation of the fluorescence quenching effect between MIPs and the PFOA concentration varied from 0 to 5 μM in combination with an ultraviolet dark-box device to achieve specific recognition of PFOA in test samples. The proposed methods enable quantitative analysis of PFOA in water samples, fulfill the requirements for real-time monitoring, and thus provide strong technical support for applications in environmental and food safety surveillance.

## 2. Experiment

### 2.1. Materials and Instruments

(3-Aminopropyl)triethoxysilane (APTES, >98%) was purchased from Tokyo Chemical Industry Co., Ltd. (Tokyo, Japan). Tetraethyl orthosilicate (TEOS, ≥99.0%) was purchased from Sigma-Aldrich (St. Louis, MO, USA). Ethanol absolute (EtOH), ammonia solution (NH_3_·H_2_O), NaCl, KCl, KNO_3_, MgCl_2_, CaCl_2_, NaSO_4_, NaOH, and HCl were purchased from Sinopharm Chemical Reagent Co., Ltd. (Shanghai, China). Sodium trifluoroacetate (C_2_F_3_NaO_2_), sodium dodecylbenesulfonate (C_12_H_25_SO_3_Na), and heptafluorobutyric acid (PFBA) were purchased from Shanghai McLean Biochemical & Technology Co., Ltd. (Shanghai, China). Perfluorobutane-1-sulphonic acid (PFSA, 98.7%) was purchased from Beijing Manhage Bio-Technology Company, Ltd. (Beijing, China). And other PFASs, including Perfluorooctanoic acid (PFOA), perfluorooctane sulfonates (PFOS), perfluorodecanoic acid (PFDA), perfluoroheptanoic acid (PFHxA), and perfluorononanoic acid (PFNA), were purchased from Dr. Ehrenstorfer (Augsburg, Europe). Humic acid (HA), Tris and PBS buffer aqueous were purchased from Beijing Solarbio Science & Technology Co., Ltd. (Beijing, China). The tap water sample, river water sample and seawater samples were collected from East China Sea Fisheries Research Institute, Chinese Academy of Fishery Sciences (Shanghai, China). Ultrapure water (18.2 MΩ·cm) was used in all the experiments, and all the reagents were used without further purification.

Transmission electron microscope (TEM) images were obtained by Thermo Fisher-Talos F200X (Thermo Fisher, Waltham, MA, USA). SHIMADZU-AXIS Supra+ (Shimadzu, Manchester, UK) spectrometer was used to record the X-ray photoelectron spectroscopy (XPS) spectra. Fourier transform infrared (FTIR) spectrum was recorded by a SHIMADZU-IRTracer-100 FTIR absorption spectrophotometer (Shimadzu, Kyoto, Japan). Zeta potential data was measured by Malvern Zetasizer Nano ZS90 (Surrey, UK). The fluorescence decay curves were measured by an FLS-980 fluorescence spectrometer (Edinburgh, UK) equipped with a 445 nm excitation laser. A UV-4100 spectrophotometer (Shimadzu, Kyoto, Japan) and an F97Pro fluorescence spectrophotometer (Lingguang, Shanghai, China) were employed to measure the UV–vis absorption and fluorescence spectra, respectively. A portable ZF-20D dark ultraviolet lightbox (Junhui Analytical Instruments, Shanghai, China) and a Huawei Mate 30 smartphone (Shenzhen Huawei Technology, Shenzhen, China) equipped with the color recognizer software were used to collect the fluorescence pictures of the test samples for G/B data acquisition.

### 2.2. Preparation of SiQDs and SiQDs@SiO_2_

Fluorescent silicon quantum dots (SiQDs) were synthesized according to a previously reported methodology [24]. Initially, an argon-saturated glycerol solution was prepared within a 100 mL flask. Subsequently, 0.3180 g of trisodium citrate dihydrate was introduced into the aforementioned flask. After intense agitation, 3 mL of APTES was gradually added drop-by-drop to the homogeneous reaction system, followed by continuous stirring for a few minutes. Finally, the prepared precursor solution was transferred to an atmospheric microwave reactor and heated at 180 °C for 15 min to synthesize SiQDs, which was indicated by the color change to dark brown. The crude solution was purified through dialysis using a membrane with a molecular weight cut-off of 1000 Da for 24 h to obtain pure SiQDs.

### 2.3. Preparation of SiQDs@SiO_2_

In total, 8 mL of SiQDs and 40 mL of anhydrous ethanol along with 100 µL of TEOS were added to a 100 mL conical flask, followed by the addition of 200 µL of NH_3_·H_2_O. The mixture was stirred at room temperature for 6 h. Subsequently, 50 µL of APTES and 30 µL of ammonia water were added to cover with a layer of silicon dioxide, and the precursor was stirred for another 8 h to produce silica-coated silicon nanoparticles (SiQDs@SiO_2_). Finally, the resulting fluorescent nanoparticles were washed three times with ethanol to remove unreacted reagents and vacuum-dried in an oven at 60 °C overnight.

### 2.4. Fabrication of MIPs and NIPs

**Scheme 1:**
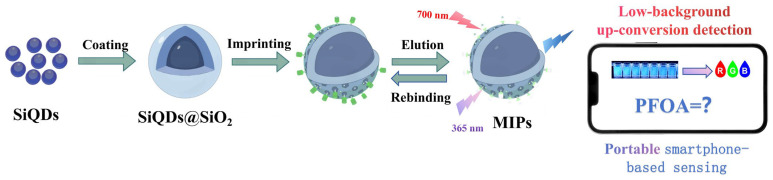
Schematic illustration of the preparation of MIPs and dual-mode detection of PFOA.

In total, 5 mg of PFOA was dissolved in 20 mL of ethanol, followed by adding 20 mg of SiQDs@SiO_2_, and ultrasonicated for 30 min to achieve the uniform dispersion. Subsequently, 45 mL of APTES was then slowly added and stirred to prefabricate for 1 h, and then 25 mL of TEOS was added and continuously stirred for another 30 min. 50 µL of NH_3_·H_2_O was added to the reaction system, which was stirred for 12 h at room temperature and centrifuged at 10,000 rpm for 15 min to collect the precipitate. After the obtained precipitate was eluted for another 12 h using methanol and centrifuged at 10,000 rpm for 15 min to remove the supernatant, the product was dried in a 60 °C vacuum oven to yield the MIPs. The preparation schematic diagram of MIPs was shown in Scheme 1. The NIPs were prepared following the same procedure but without addition of the template molecule (i.e., PFOA).

### 2.5. Up-Conversion Fluorescence Detection of PFOA

Detection of PFOA was performed as follows. MIPs or NIPs suspension, various concentrations of PFOA and Tris (pH 8) buffer were added to the glass tubes. After thorough mixing, the mixtures were incubated for 15 min to detect PFOA. All experiments were performed in triplicate to ensure precision. Fluorescence spectra were acquired at an excitation wavelength of 710 nm, a voltage of 1000 V, an excitation slit width and emission slit width of 20 nm.

Generally, the alteration in fluorescence intensity stems from the interaction between fluorescent probe molecules and fluorescence quenchers, which typically originates from static quenching or dynamic quenching. The quenching kinetics can be characterized by the Stern–Volmer equation [29]:

(1) $$\frac{F_{0}}{F}=K_{\mathrm{SV}}\left[Q\right]+1$$

where F_0_ and F denote the fluorescence intensities of MIPs or NIPs in the absence and presence of PFOA, respectively, Ksv represents the Stern–Volmer quenching constant, and [Q] signifies the concentration of PFOA.

### 2.6. Smartphone-Enabled Detection of PFOA

A smartphone-integrated sensing platform was devised for the rapid and portable detection of PFOA. The test solutions were added to one row or column of the 96-well plate for side-view photography, subsequently placed within an ultraviolet (UV) dark chamber, along with the smartphone in the outer side of the dark box, then closed the dark box and turned on the switch of the 365 nm UV lamp to excite the tested samples, followed by captured fluorescence photos by the smartphone’s camera, which were analyzed by color recognition software to extract the corresponding RGB values, converting to G/B value to analyze the correlation between G/B value and PFOA concentration.

## 3. Results and Discussion

### 3.1. Preparation and Characterization of MIPs

Figure S1 depicts the influence of the volume ratio of APTES to TEOS on the fluorescence variation (F_0_/F-1) of MIPs. Five groups of materials with ratios spanning from 9:1 to 9:9 were separately prepared. In comparison with other groups, the fluorescence change in MIPs achieved the maximum value as the volume ratio of APTES to TEOS was set to 9:5. As presented in Figure S2, the adsorption capacity of MIPs is evidently superior to that of NIPs, and the imprinting factor was calculated to be 4.22.

**Figure 1:**
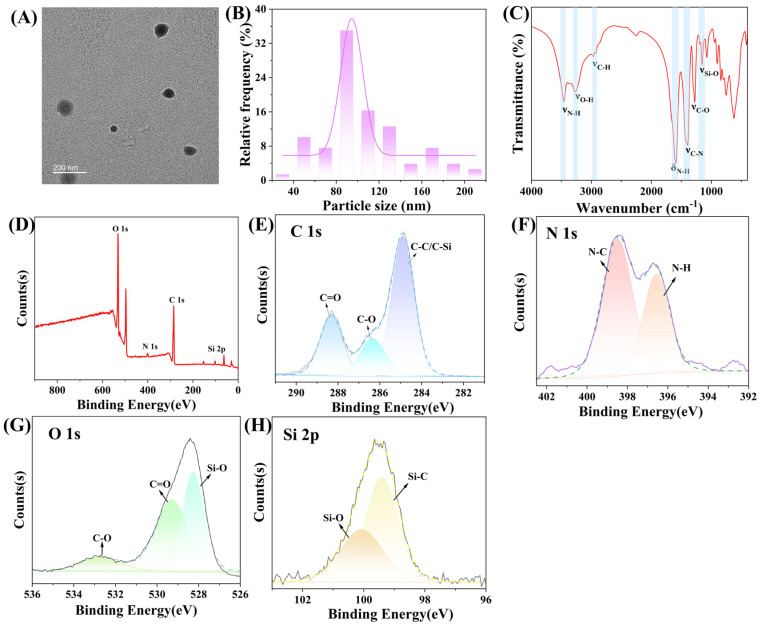
(**A**) TEM images. (**B**) Particle size distribution. (**C**) FTIR spectrum. (**D**) XPS survey spectrum. (**E**) C1s spectrum. (**F**) N1s spectrum. (**G**) O1s spectrum. (**H**) Si 2p spectrum of MIPs.

The structural characteristics of the as-prepared MIPs were investigated by TEM, FTIR, and XPS. The TEM image reveals the dimensions of the fabricated MIPs. As depicted in Figure 1A, MIPs presented a multitude of spherical particles, and a thin and luminous SiO_2_ imprint layer covered the surface of MIPs, which signified the successful synthesis of MIPs. Figure 1B illustrates the particle size distribution of MIPs, and the Gaussian fitting curve indicates that the average size of MIPs was approximately 94 nm. The FTIR spectrum verified the existence of surface functional groups on the MIPs. As displayed in Figure 1C, the peaks at 3458 cm^−1^, 3270 cm^−1^ and 2968 cm^−1^ corresponded to the stretching vibrations of the N-H, O-H, and C-H bonds, respectively [30]. The peak at 1593 cm^−1^ was attributed to the bending vibration of N-H [31]. The peaks at 1403 cm^−1^, 1278 cm^−1^ and 1155 cm^−1^ were assigned to the stretching vibrations of the C-N, C-O and Si-O bonds, respectively [32,33]. Results also corroborated the successful formation of MIPs.

XPS further verified the elemental composition of the MIPs. As observed in Figure 1D, the surface of MIPs was predominantly composed of C, N, O, and Si elements. In the C 1s profile of the MIPs (Figure 1E), the peaks at 284.7 eV, 286 eV, and 288 eV were ascribed to C-C/C-Si, C-O, and C=O bonds [34], respectively. The N 1s spectrum consisted of peaks at 398.5 and 396 eV, indicating the presence of N-C and N-H bonds [35] (Figure. 1F). In the O 1s spectrum, the peaks at 528, 529, and 532.9 eV were assigned to Si-O, C=O, and C-O bonds, respectively [36] (Figure 1G). In the Si 2p spectrum, the peak at 99.3 eV was attributed to the Si-C bond, whereas the peak at 101.3 eV corresponded to the Si-O bond [37] (Figure 1H). The XPS results were in accordance with those obtained from the FTIR spectrum, further validating the successful synthesis of the MIPs.

### 3.2. Optical Properties of MIPs

**Figure 2:**
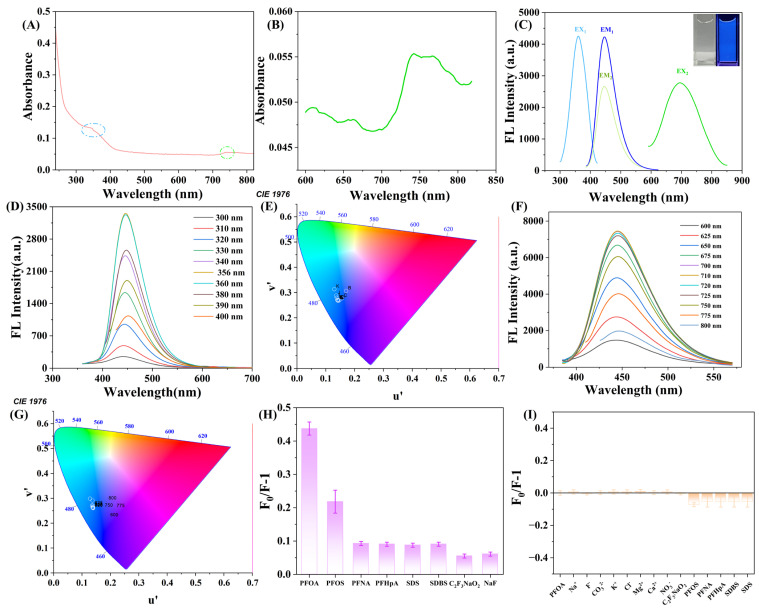
Optical properties of the prepared MIPs: (**A**,**B**) Ultraviolet-visible absorption spectra; (**C**) fluorescence excitation spectra (EX_1_ and EX_2_), and emission spectra (EM_1_ and EM_2_), Inset: photographs of the MIPs aqueous solution under daylight (left) and 365 nm UV light (right). (**D**) Fluorescence emission spectra and (**E**) CIE diagram with excitation wavelengths evenly spaced from 300 to 400 nm. (**F**) Up-conversion fluorescence spectra and (**G**) CIE diagram with excitation wavelengths varied from 600 to 800 nm. (**H**) Selectivity evaluations. (**I**) Anti-interference assessments.

Figure 2 illustrates the optical properties of the synthesized MIPs. As depicted in Figure 2A, the MIPs displayed a broad absorption band centered at approximately 356 nm (blue dashed circle) and a weak absorption peak at 730 nm (green dashed circle) in the ultraviolet-visible (UV–Vis) absorption spectra (Figure 2B). As observed in Figure 2C, MIPs exhibited correspondingly two distinct excitation peaks at 360 nm (blue line, Ex_1_) and 690 nm (green line, Ex_2_), along with a symmetric emission peak at 447 nm (blue line of Em_1_, green line of Em_2_). Additionally, the aqueous solution of MIPs appeared transparent under ambient daylight and emitted intense blue fluorescence upon excitation by 365 nm UV light, which rendered it suitable for subsequent smartphone-integrated fluorescence sensing applications (inset of Figure 2C). Meanwhile, the emission wavelength of the MIPs remained nearly constant and located in the blue region with the excitation wavelength spanning 300–400 nm (Figure 2D,E). Moreover, as illustrated in Figure 2F,G, the MIPs emitted blue fluorescence at 447 nm and were also located in the blue region with excitation wavelength in the range 600–800 nm, which demonstrated excellent up-conversion fluorescence performance, indicating the potential for developing low-background methods.

A diverse range of coexisting compounds (Cl^−^, F^−^, NO_3_^−^, CO_3_^2−^, Na^+^, K^+^, Ca^2+^, Mg^2+^ and several analogs) were utilized to examine the selectivity and anti-interference capacity of MIPs under the identical detection conditions. As shown in Figure 2H, the fluorescence change in MIPs in response to PFOA was significantly higher than that for other analogs, demonstrating the specific recognition ability of MIPs towards PFOA. Furthermore, the fluorescence change in MIPs for PFOA remained largely unaffected upon the addition of other analogs or ions, implying the good anti-interference performance (Figure 2I). The excellent selectivity and anti-interference ability of MIPs towards PFOA stem from the formed imprinted cavities, which demonstrate a perfect match between their size, pore size, molecular length and configuration of PFOA. Consequently, only PFOA is compatible with the cavity size and can enter the cavity, whereas other non-target molecules are excluded owing to size discrepancies.

### 3.3. Fluorescence Quenching Mechanism Between MIPs and PFOA

**Figure 3:**
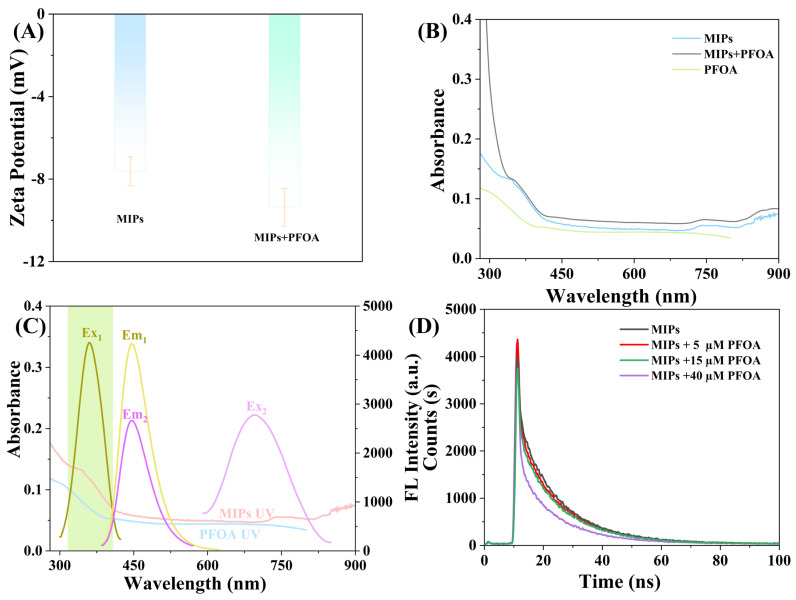
(**A**) Zeta potentials of the MIPs and MIPs + PFOA system. (**B**) UV–Vis absorption spectra of the MIPs, PFOA, and MIPs + PFOA system. (**C**) UV–Vis absorption spectra of MIPs and PFOA, along with excitation (Ex_1_ and Ex_2_) and emission (Em_1_ and Em_2_) spectra of MIPs. (**D**) Fluorescence decay curves of the MIPs and MIPs + PFOA system.

The interactions between PFOA and the surface functional groups of the MIPs lead to the fluorescence quenching of MIPs, which was explored through zeta potential analysis, UV–Vis absorption spectroscopy, and fluorescence lifetime decay measurements [38]. The surface of MIPs typically features polar groups, such as hydroxyl groups, which can easily generate electrostatic interactions or hydrogen bonds with the target molecule, altering the charge distribution on the surface of the composite material, leading to a change in the zeta potential value. As presented in Figure 3A, the zeta potential of the MIPs changed from −6.87 mV to −10.2 mV subsequent to the addition of PFOA, which confirmed the interaction (electrostatic interactions, hydrogen bonds, etc.) between MIPs and PFOA. As shown in Figure 3B, comparing the UV–Vis absorption spectra of MIPs (blue line) and PFOA (green line), no new peak was observed in the UV–Vis absorption spectrum of MIPs + PFOA mixture solution (gray line), which indicated the absence of static quenching and the presence of dynamic quenching [39]. As observed in Figure 3C, the UV–Vis absorption spectrum of PFOA (blue line) overlapped (green area)_ with both the UV–Vis absorption spectrum (red line) and excitation spectrum (Ex_1_ and Ex_2_) of MIPs, suggesting that the fluorescence quenching primarily originated from photoinduced electron transfer (PET) [40,41]. The strong electronegativity of fluorine atoms can attract electrons on the surface of MIPs, alter the electron cloud distribution of MIPs, and significantly increase the probability of non-radiative transitions of excited state electrons in MIPs, leading to fluorescence quenching. In contrast, there was negligible overlap between the UV–Vis absorption spectrum of PFOA (blue line) and the emission spectrum of MIPs (Em_1_ and Em_2_), eliminating the possibility of fluorescence resonance energy transfer [42] (FRET). The fluorescence decay curves further clarified the interaction between PFOA and MIPs. As shown in Figure 3D, the fluorescence decay curves of MIPs gradually declined as the concentration of PFOA increased from 0 to 5 µM, 15 µM and 40 µM, confirming that the dynamic quenching effect contributed to the feasibility of MIPs as fluorescent probes for PFOA detection [43]. As shown in Figure 3C, the fluorescence decay curves of MIPs gradually declined and the fitted mean lifetime (τ) decreased from 14.76 ns, 14.20 ns, 14.14 ns, and ultimately to 14.10 ns as the concentration of PFOA increased from 0 to 5 µM, 15 µM to 40 µM, confirming that the dynamic quenching effect contributed to the feasibility of MIPs as fluorescent probes for PFOA detection [43].

### 3.4. Low-Background Up-Conversion Fluorescence Method and Portable Smartphone-Sensing Platform Method for the Detection of PFOA

Owing to the excellent optical properties, selectivity and anti-interference ability, the prepared MIPs materials could be employed for the detection of PFOA. In order to achieve a favorable detection response, the effects of detection conditions on fluorescence intensity of MIPs were optimized, including pH, material dosage, equilibrium time and so on. As shown in Figure S3A–C, the change in fluorescence intensity for MIPs differed under different pH conditions. And MIPs demonstrated optimal detection performance at a pH of 8.0 in PBS and Tris buffers. Considering that environmental water contains numerous heavy metals that could complex with phosphate and impact the detection effect, the Tris buffer was selected to mitigate the influence of pH. As shown in Figure S3D, the change in fluorescence intensity of the MIP + PFOA system initially increased with the MIP dosage, ranging from 0.1 to 0.6 mg·mL^−1^, and then decreased as the MIP dosage further increased to 0.8 mg·mL^−1^. Therefore, a MIP dosage of 0.6 mg·mL^−1^ was chosen for the fluorescence determination of PFOA. As illustrated in Figure S3E, the fluorescence intensity of the MIP+PFOA solution gradually reached a stable equilibrium state after mixing for 15 min. Consequently, the optimal detection time was determined to be 15 min after the sample was allowed to stand for reaction equilibrium.

**Figure 4:**
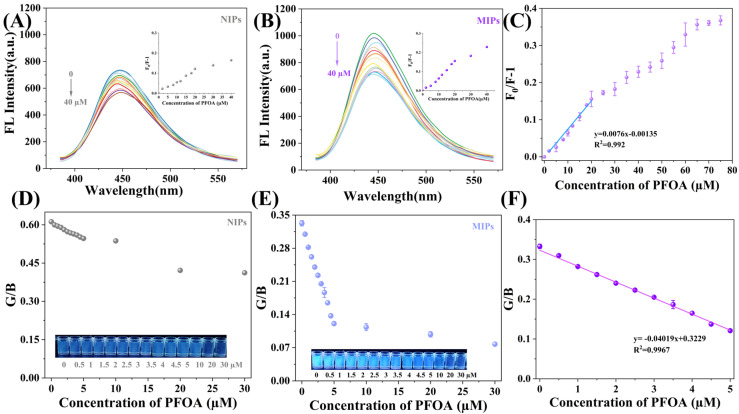
Fluorescence spectra of NIPs (**A**) and MIPs (**B**) in the presence of varying concentrations of PFOA from 0 to 40 μM, insets: plot of F_0_/F-1 against PFOA concentration for NIPs and MIPs. (**C**) Relationship between F_0_/F-1 and PFOA concentration ranging from 0 to 80 μM. Relationship between G/B value derived from smartphone-captured fluorescence images of NIPs (**D**) or (**E**) MIPs and PFOA at different concentration ranges. Insets: Fluorescence images of NIPs or MIPs under 365 nm UV light irradiation in the presence of PFOA at diverse concentrations. (**F**) Linear correlation between G/B value from fluorescence images of MIPs and PFOA concentration.

Under the optimal detection conditions, an investigation was conducted on the up-conversion fluorescence and smartphone-based detection methods of PFOA. Both of the fluorescence intensity of NIPs (Figure 4A) and MIPs (Figure 4B) gradually decreased with the PFOA concentration increasing from 0 to 40 µM. Significantly, the change in fluorescence intensity (F_0_/F-1) of MIPs was markedly superior to that of the NIPs (insets of Figure 4A,B), which indicated that the prepared MIPs exhibited a favorable imprinting effect compared to that of NIPs. Simultaneously, F_0_/F-1 increased as PFOA concentration rose from 0 to 80 µM, and a good linear correlation between F_0_/F-1 and the PFOA concentration within the range of 2–20 µM with a correlation coefficient (R^2^) of 0.992 and a limit of detection (LOD) of 37.5 nM (S/N = 3), which was comparable to those reported in the previous literature (Table 1), indicating that the as-prepared MIPs could serve as a simple and feasible nano-fluorescent probe sensor for low-background detection of PFOA.

Furthermore, a smartphone-based technique integrated with a UV dark box device was developed for the portable identification of PFOA. As shown in Figure 4D,E, compared to that of NIPs, the brightness of the fluorescence images of MIPs (insets) gradually diminished, and G/B values of MIPs markedly decreased as the PFOA concentration increased from 0 to 30 µM, which also suggested the satisfactory imprint effect of the prepared MIPs. Moreover, Figure 4F reveals that a good linear relationship exists between the G/B value and PFOA concentration in the range of 0–5 µM (R^2^ = 0.9967, LO = 73.9 nM). Thus, results confirm that the established smartphone-integrated MIP-based molecularly imprinted fluorescent sensing method is feasible for the on-site detection of PFOA in aqueous samples.

### 3.5. Application in Real Sample Analysis

To evaluate the accuracy, reproducibility, and practicality of the established methods, spike addition experiments were carried out in actual environmental water and food samples, including tap water, seawater, milk, and orange juice. As presented in Table 2, in tap water spiked with 5 μM PFOA, the established up-conversion fluorescence method measured 5.72 μM, which was close to 5.12 μM obtained by the standard HPLC-MS/MS method. Moreover, similar agreement was observed at other spiking levels in tap water and orange juice, verifying the accuracy of the proposed up-conversion fluorescence method. The recovery rates ranged from 86.1% to 114.4%, with relative standard deviations (RSDs) between 0.6% and 7.6%, indicating that the established MIP-based PFOA detection method demonstrated accuracy, reproducibility, and feasibility. Meanwhile, in tap water spiked with 1.5 μM PFOA, the developed smartphone-based method tested 1.55 μM, which was identical to 1.36 μM acquired by the standard HPLC-MS/MS method. Furthermore, the identical results were also demonstrated at other spiking levels in tap water and orange juice, verifying the accuracy of the proposed smartphone-based method. The recovery rates of the smartphone-based intelligent sensing platform were within the range of 87.3% to 117.3%, with RSDs from 0.6% to 5.9%, which also verified the accuracy and reliability of the established smartphone-integrated fluorescent sensing system for PFOA detection.

Subsequently, this method was applied to the detection of PFOA in real samples. No PFOA was detected in the collected tap water, river water, seawater, and food samples, which was consistent with previous research findings [51], suggesting that the tested environmental water bodies and food samples are relatively safe and currently pose no risks to human health.

## 4. Conclusions

In summary, we developed dual-mode methods including a low-background up-conversion detection method and a portable rapid smartphone-sensing method for PFOA detection in environmental and food samples based on the silicon quantum dot molecularly imprinted polymers (MIPs). The synthesized MIPs presented remarkable advantages including abundant surface functional groups, excellent fluorescence, unique up-conversion fluorescence, good selectivity and anti-interference ability. This design synergistically combines the advantages of the excellent up-conversion fluorescence properties of the SiQDs, minimizing autofluorescence from complex sample matrices, and outstanding selectivity for PFOA recognition of MIPs. It was dynamic quenching evidenced by optical spectra and fluorescence decay curves that was responsible for the fluorescence quenching of MIPs with the presence of PFOA. Under optimal experimental conditions, a favorable low-background up-conversion fluorescence detection method using the prepared MIPs was established within the linear range of 2–20 μmol/L for PFOA concentration and a detection limit of 37.5 nmol/L. Moreover, a rapid-response and portable smartphone-based sensing method by analyzing the G/B value of MIP fluorescence images was identified to detect PFOA concentration in the range of 0–5 μmol/L with a detection limit of 73.9 nmol/L, facilitating rapid, on-site analysis. Recovery tests conducted on real environmental and food samples verified the accuracy, reproducibility, and feasibility of the established methods. This research offered a novel dual-mode strategy for the low-background and portable detection based on intelligent sensing MIPs, enabling direct analysis of complex samples with minimal pretreatment and leveraging ubiquitous smartphone technology, significantly reducing reliance on sophisticated laboratory instrumentation. It highlights their potential application value in the fields of environmental and food monitoring and assessment.

## Figures and Tables

**Table 1:** Comparison of the performance of different fluorescent materials in detecting PFOA.

Materials	Linear Ranges	LOD	References
cp. GFP/hLFABP	0–50 µM	797 nM	[44]
Alg/GO-FPI-OFS	0–4.83 nM	0.97 nM	[45]
PVDF-OFS	0–144.9 µM	12.1 µM	[46]
bubble-nucleation-based electrochemical	241.5 nM–241.5 µM	72.5 nM	[47]
CNF-Cu/C-SPEF	1–100 μM	133 nM	[48]
AFFFs-MB-MIP	10 µM–10 mM	100 nM	[49]
PFAEI	0–460 nM	15 nM	[50]
ES-tetraphenyl derivatives	1.5–10.5 μM	4.75 nM	[17]
SiQDs–MIPs	2–20 μM 0–5 μM	37.5 nM 73.9 nM	This work

**Table 2:** Application of the developed up-conversion fluorescence method and smartphone-based intelligent sensing platform for PFOA detection in spiked real samples.

Analytes	Up-Conversion Fluorescence Method				HPLC-MS/MS Method	Smartphone-Based Method				HPLC-MS/MS Method
	Spiked (µM)	Found (µM)	Recovery (%)	RSD (%)	Found (µM)	Spiked (µM)	Found (µM)	Recovery (%)	RSD (%)	Found (µM)
Tap water	0	*N.F.*	-	-	0.016	0 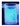	*N.F.*	-	-	0.016
	5	5.72 ± 0.028	114.4	1.2	5.12	1.5 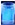	1.55 ± 0.045	103.3	1.4	1.36
	10	9.24 ± 0.088	92.4	1.4	8.02	2.5 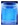	2.58 ± 0.088	103.2	1.5	2.60
	15	12.92 ± 0.017	86.1	3.4	11.94	3.5 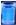	3.49 ± 0.062	99.7	1.4	3.26
Sea water	0	*N.F.*	-	-	-	0 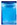	*N.F.*	-	-	-
	5	5.48 ± 0.090	109.6	2.7	-	1.5 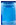	1.53 ± 0.094	102.0	1.8	-
	10	10.67 ± 0.039	106.7	1.8	-	2.5 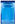	2.74 ± 0.097	109.6	2.9	-
	15	16.29 ± 0.066	108.6	2.8	-	3.5 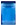	3.21 ± 0.014	91.7	1.5	-
Milk	0	*N.F.*	-	-	-	0 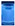	*N.F.*	-	-	-
	5	4.34 ± 0.087	86.8	7.6	-	1.5 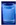	1.76 ± 0.077	117.3	5.9	-
	10	11.25 ± 0.020	112.5	1.2	-	2.5 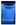	2.67 ± 0.082	106.8	1.8	-
	15	16.64 ± 0.044	110.9	2.9	-	3.5 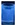	3.46 ± 0.011	99.9	2.1	-
Orange juice	0	*N.F.*	-	-	0.006	0 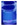	*N.F.*	-	-	0.006
	5	4.91 ± 0.032	98.2	0.6	4.98	1.5 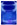	1.31 ± 0.028	87.3	1.2	1.54
	10	9.83 ± 0.075	98.3	1.8	9.82	2.5 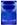	2.67 ± 0.069	106.8	0.8	2.64
	15	12.94 ± 0.096	86.3	3.2	12.3	3.5 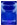	3.74 ± 0.054	106.9	0.6	3.62

*N.F.*: not found.

## Data Availability

The original contributions presented in this study are included in the article/Supplementary Material. Further inquiries can be directed to the corresponding author.

## Supplementary Materials

The following supporting information can be downloaded at https://www.mdpi.com/article/10.3390/foods15020331/s1, Figure S1: The effect of the ratio of APTES and TEOS on the fluorescence of MIPS; Figure S2: The adsorption capacity of MIPs and NIPs for PFOA. Figure S3: (A) Effect of PBS buffer at different pH values on the fluorescence intensity of the reaction system; (B) Effect of Tris buffer at different pH values on the fluorescence intensity of the reaction system; (C) Effect of Tris and PBS buffers on the fluorescence intensity of the reaction system at pH 8.0; (D) Variation of the fluorescence intensity of the reaction system with different MIP dosages; (E) Influence of equilibrium time on the fluorescence intensity of the reaction system.

## References

[1] de Souza B.B., Perera D.C., Teymourian T., Meegoda J.N. (2025). Mechanism of sonolytic PFAS degradation into inorganic products: A combined reactive molecular dynamics and density functional theory approaches. J. Colloid Interface Sci..

[2] Perera D.C., Meegoda J.N. (2024). PFAS: The journey from wonder chemicals to environmental nightmares and the search for solutions. Appl. Sci..

[3] Tian Y., Yao Y., Chang S., Zhao Z., Zhao Y., Yuan X., Wu F., Sun H. (2018). Occurrence and phase distribution of neutral and ionizable per-and polyfluoroalkyl substances (PFASs) in the atmosphere and plant leaves around landfills: A case study in Tianjin, China. Environ. Sci. Technol..

[4] Dimitrakopoulou M.E., Karvounis M., Marinos G., Theodorakopoulou Z., Aloizou E., Petsangourakis G., Papakonstantinou M., Stoitsis G. (2024). Comprehensive analysis of PFAS presence from environment to plate. npj Sci. Food.

[5] Ryu H., Li B., De Guise S., McCutcheon J., Lei Y. (2021). Recent progress in the detection of emerging contaminants PFASs. J. Hazard. Mater..

[6] Messmer M.F., Siegel L., Locwin B. (2024). Global manufacturer concealed hazards of PFAS releases for decades. Curr. Opin. Green Sustain. Chem..

[7] Shahi S., Winquist A., Troeschel A.N., Teras L.R., Campbell P.T., Flanders W.D., Goodman M. (2025). A Case-Cohort Study of the Association between Per-and Polyfluoroalkyl Substances (PFAS) and Breast Cancer among Participants in the American Cancer Society’s Cancer Prevention Study-II. Environ. Res..

[8] Tokranov A.K., Ransom K.M., Bexfield L.M., Lindsey B.D., Watson E., Dupuy D.I., Zeighami E.A., Campagnolo E.R., LeBlanc D.R. (2024). Predictions of groundwater PFAS occurrence at drinking water supply depths in the United States. Science.

[9] Breshears L.E., Mata-Robles S., Tang Y., Baker J.C., Reynolds K.A., Yoon J.-Y. (2023). Rapid, sensitive detection of PFOA with smartphone-based flow rate analysis utilizing competitive molecular interactions during capillary action. J. Hazard. Mater..

[10] Domingo J.L., Nadal M. (2019). Human exposure to per- and polyfluoroalkyl substances (PFAS) through drinking water: A review of the recent scientific literature. Environ. Res..

[11] Pérez F., Nadal M., Navarro-Ortega A., Fàbrega F., Domingo J.L., Barceló D., Farré M. (2013). Accumulation of perfluoroalkyl substances in human tissues. Environ. Int..

[12] Fu J., Gao Y., Cui L., Wang Y., Zhang A., Sun J., Liu W., Li G., Sun Z., Jiang G. (2016). Occurrence, temporal trends, and half-lives of perfluoroalkyl acids (PFAAs) in occupational workers in China. Sci. Rep..

[13] Fang C., Megharaj M., Naidu R. (2015). Chemical oxidization of some AFFFs leads to the formation of 6:2FTS and 8:2FTS. Environ. Toxicol. Chem..

[14] Robel A.E., Marshall K., Dickinson M., Lunderberg D., Butt C., Peaslee G., Stapleton H.M. (2017). Closing the mass balance on fluorine on papers and textiles. Environ. Sci. Technol..

[15] Poothong S., Boontanon S.K., Boontanon N. (2012). Determination of perfluorooctane sulfonate and perfluorooctanoic acid in food packaging using liquid chromatography coupled with tandem mass spectrometry. J. Hazard. Mater..

[16] Zacs D., Bartkevics V. (2016). Trace determination of perfluorooctane sulfonate and perfluorooctanoic acid in environmental samples (surface water, wastewater, biota, sediments, and sewage sludge) using liquid chromatography-Orbitrap mass spectrometry. J. Chromatogr. A.

[17] Yao P., Ou J., Zhao D., Zhou S., Wang Q., Feng Y., Meng X. (2025). An AIE-activated fluorescent sensor for one-step concurrent detection and bioimaging of perfluorooctanoic acid and perfluorooctane sulfonate in vitro and in vivo. J. Hazard. Mater..

[18] Chen Q., Liu J., Liu S., Zhang J., He L., Liu R., Jiang H., Han X., Zhang K. (2023). Visual and rapid detection of nerve agent mimics in gas and solution phase by a simple fluorescent probe. Anal. Chem..

[19] Zhang J., Hou J., Zhang K., Zhang R., Geng J., Wang S., Zhang Z. (2022). Integration of quantum dots with Zn_2_GeO_4_ nanoellipsoids to expand the dynamic detection range of uranyl ions in fluorescent test strips. J. Hazard. Mater..

[20] Geng X., Li Z., Hu Y., Liu H., Sun Y., Meng H., Wang Y., Qu L., Lin Y. (2018). One-pot green synthesis of ultrabright N-doped fluorescent silicon nanoparticles for cellular imaging by using ethylenediaminetetraacetic acid disodium salt as an effective reductant. ACS Appl. Mater. Interfaces.

[21] Qin Y.T., Peng H., He X.W., Li W.Y., Zhang Y.K. (2019). Highly effective drug delivery and cell imaging using fluorescent double-imprinted nanoparticles by targeting recognition of the epitope of membrane protein. Anal. Chem..

[22] Ma Y.J., Li S., Qin Y.T., He X.W., Li W.Y., Zhang Y.K. (2023). Multimode sensing platform based on turn-on fluorescent silicon nanoparticles for monitoring of intracellular pH and GSH. Anal. Chem..

[23] Cao L., Wang X., Meziani M.J., Lu F.S., Wang H.F., Luo P.G., Lin Y., Harruff B.A., Veca L.M., Murray D. (2007). Carbon dots for multiphoton bioimaging. J. Am. Chem. Soc..

[24] Ye H.L., Cai S.N.J., Li S., He X.W., Li W.Y., Li Y.H., Zhang Y.K. (2016). One-pot microwave synthesis of water-dispersible, high fluorescence silicon nanoparticles and their imaging applications in vitro and in vivo. Anal. Chem..

[25] Brown S. (2008). Photodynamic Therapy: Two photons are better than one. Nat. Photonics.

[26] Wang D., Zhu L., Pu Y., Wang J.X., Chen J.F., Dai L.M. (2017). Transferrin-coated magnetic upconversion nanoparticles for efficient photodynamic therapy with near-infrared irradiation and luminescence bioimaging. Nanoscale.

[27] Çelik H., Caf B.B., Çebi G. (2025). Smartphone-assisted biosensors in point-of-care diagnostics: Integration, applications, and future challenges. Chem. Pap..

[28] Rajendran V.K., Bakthavathsalam P., Bergquist P.L., Anwar S. (2021). Smartphone technology facilitates point-of-care nucleic acid diagnosis: A beginner’s guide. Crit. Rev. Clin. Lab. Sci..

[29] Kou M., Qin F., Wang Y., Zhang X., Li L., Hu Z., Zhao H., Zhang Z. (2023). Effects of excitation power density on the Stern–Volmer constant measurement. Opt. Lett..

[30] Chun H.J., Ocola E.J., Laane J. (2016). Vapor-phase infrared and Raman spectra and ab-initio calculations of the axial and equatorial forms of cyclohexane-d1 and d11. J. Mol. Spectrosc..

[31] Calabrò E., Currò M., Caccamo M.T., Lentile R., Magazù S. (2020). Competition between N–H bending vibration and α-helix polarization under 50 Hz magnetic field in SH-SY5Y neuronal-like cells. Spectrosc. Lett..

[32] Hatmanto A.D., Kita K. (2020). Physical analysis of remained oxidation byproducts as the origins of lattice distortion at the surface of 4H-SiC by Fourier-transform infrared spectroscopy. Jpn. J. Appl. Phys..

[33] Lobanok M.V., Mukhammad A.I., Gaiduk P.I. (2022). Structural and Optical Properties of SiC/Si Heterostructures Obtained Using Rapid Vacuum-Thermal Carbidization of Silicon. J. Appl. Spectrosc..

[34] Mohai M., László K., Bertóti I. (2018). Reduction and covalent modification of graphene-oxide by nitrogen in glow discharge plasma. Surf. Interface Anal..

[35] Yadav I., Dutta S., Pandey A., Yadav N., Goyal A., Chatterjee R. (2020). Evolution of TiOx-SiOx nano-composite during annealing of ultrathin titanium oxide films on Si substrate. Ceram. Int..

[36] Liu S., Xin Z.J., Lei Y.J., Yang Y., Yan X.Y., Lu Y.B., Li C.B., Wang H.Y. (2017). Thin copper-based film for efficient electrochemical hydrogen production from neutral aqueous solutions. ACS Sustain. Chem. Eng..

[37] Ootani Y., Xu J., Takahashi N., Kanda N., Nakamura T., Morita N., Nakayama Y., Kubo M., Koganezawa S., Sugimura J. (2020). Self-formed double tribolayers play collaborative roles in achieving superlow friction in an aqueous environment. J. Phys. Chem. C.

[38] Zaman A.C., Kaya F., Kaya C. (2020). A study on optimum surfactant to multiwalled carbon nanotube ratio in alcoholic stable suspensions via UV–Vis absorption spectroscopy and zeta potential analysis. Ceram. Int..

[39] de Carvalho Bertozo L., Fernandes A.J.F.C., Yoguim M.I., Pereira E., Gonçalves D., Sineli P.E., da Silva R.A., Itri R. (2020). Entropy-driven binding of octyl gallate in albumin: Failure in the application of temperature effect to distinguish dynamic and static fluorescence quenching. J. Mol. Recognit..

[40] Arabi M.S., Karami C., Taher M.A., Ghaemi F., Shamsipur M. (2020). Fluorescence detection of laccases activity by the photoinduced electron transfer (PET) process. JBIC J. Biol. Inorg. Chem..

[41] Luo L., Jia B.Z., Wei X.Q., Wang J., Liu Y., Wang H., Wang S. (2021). Development of an inner filter effect-based fluorescence immunoassay for the detection of acrylamide using 9-xanthydrol derivatization. Sens. Actuators B Chem..

[42] Halter O., Plenio H. (2017). Fluorescence resonance energy transfer (FRET) for the verification of dual gold catalysis. Chem. Commun..

[43] Kumar A.T.N., Rice W.L., López J.C., Kulkarni R.A., Pudavar H.E., Cai Z., Achilefu S. (2016). Substrate-based near-infrared imaging sensors enable fluorescence lifetime contrast via built-in dynamic fluorescence quenching elements. ACS Sens..

[44] Mann M.M., Berger B.W. (2023). A genetically-encoded biosensor for direct detection of perfluorooctanoic acid. Sci. Rep..

[45] Faiz F., Cran M.J., Zhang J., Muthukumaran S., Sidiroglou F. (2023). Graphene Oxide Doped Alginate Coated Optical Fiber Sensor for the Detection of Perfluorooctanoic Acid in Water. IEEE Sens. J..

[46] Faiz F., Baxter G., Collins S., Sidiroglou F., Cran M. (2020). Polyvinylidene fluoride coated optical fibre for detecting perfluorinated chemicals. Sens. Actuators B Chem..

[47] Ranaweera R., Ghafari C., Luo L. (2019). Bubble-Nucleation-Based Method for the Selective and Sensitive Electrochemical Detection of Surfactants. Anal. Chem..

[48] Hou C., Chen F., Cheng D., Zou S., Wang J., Shen M., Wang Y. (2024). MOFs-derived Cu/Carbon membrane as bi-functional catalyst: Improving catalytic activity in detection and degradation of PFOA via regulation of reactive Cu species. Chem. Eng. J..

[49] Fang C., Chen Z., Megharaj M., Naidu R. (2016). Potentiometric detection of AFFFs based on MIP. Environ. Technol. Innov..

[50] Sahu S.P., Kole S., Arges C.G., Ranjan M. (2022). Rapid and Direct Perfluorooctanoic Acid Sensing with Selective Ionomer Coatings on Screen-Printed Electrodes under Environmentally Relevant Concentrations. ACS Omega.

[51] Grung M., Hjermann D.Ø., Rundberget T., Bæk K., Thomsen C., Knutsen K.H., Smastuen Haug L. (2024). Low levels of per-and polyfluoroalkyl substances (PFAS) detected in drinking water in Norway, but elevated concentrations found near known sources. Sci. Total Environ..

