# Supplementary material for: Dual-Mode Detection of Perfluorooctanoic Acid Using Up-Conversion Fluorescent Silicon Quantum Dots–Molecularly Imprinted Polymers and Smartphone Sensing
Source: Foods. 2026 Jan 16;15(2):331. doi: 10.3390/foods15020331 (PMC12841523; doi:10.3390/foods15020331)
Supplement: Supplementary file 1 [file foods-15-00331-s001.zip › foods-4052557-supplementary.pdf]

Supporting information

Dual-Mode Detection of Perfluorooctanoic Acid using Upconversion Fluorescent Silicon Quantum Dots Molecularly Imprinted Polymers and Smartphone Sensing

Hongli Ye[1,*], Xinran Wang[1, 2], Xiangqian Xu[1, 2], Hongyang Xu[3], Rui Yuan[1] , Ping Cheng[3]

[1]Laboratory of Aquatic Product Quality, Safety and Processing, Key Laboratory of Oceanic and Polar Fisheries, Ministry of Agriculture and Rural Affairs, East China Sea Fisheries Research Institute, Chinese Academy of Fishery Sciences, Shanghai 200090, P. R. China (Dr. H.Y. yehongli@ecsf.ac.cn; X.W. wangxinran6000@163.com; X.X. 18204876676@163.com; R.Y. yuanrui@ecsf.cn;)

[2]College of Food Science and Engineering, Dalian Ocean University, Dalian 116023, P. R. China (X.W. wangxinran6000@163.com; X.X. 18204876676@163.com;)

[3]School of Materials and Chemistry, University of Shanghai for Science and Technology, Shanghai 200093, P. R. China (H.X. 233393208@st.usst.edu.cn; Dr. P. C. chengp@usst.edu.cn)

* Corresponding author: yehongli@ecsf.ac.cn (H. Y.)

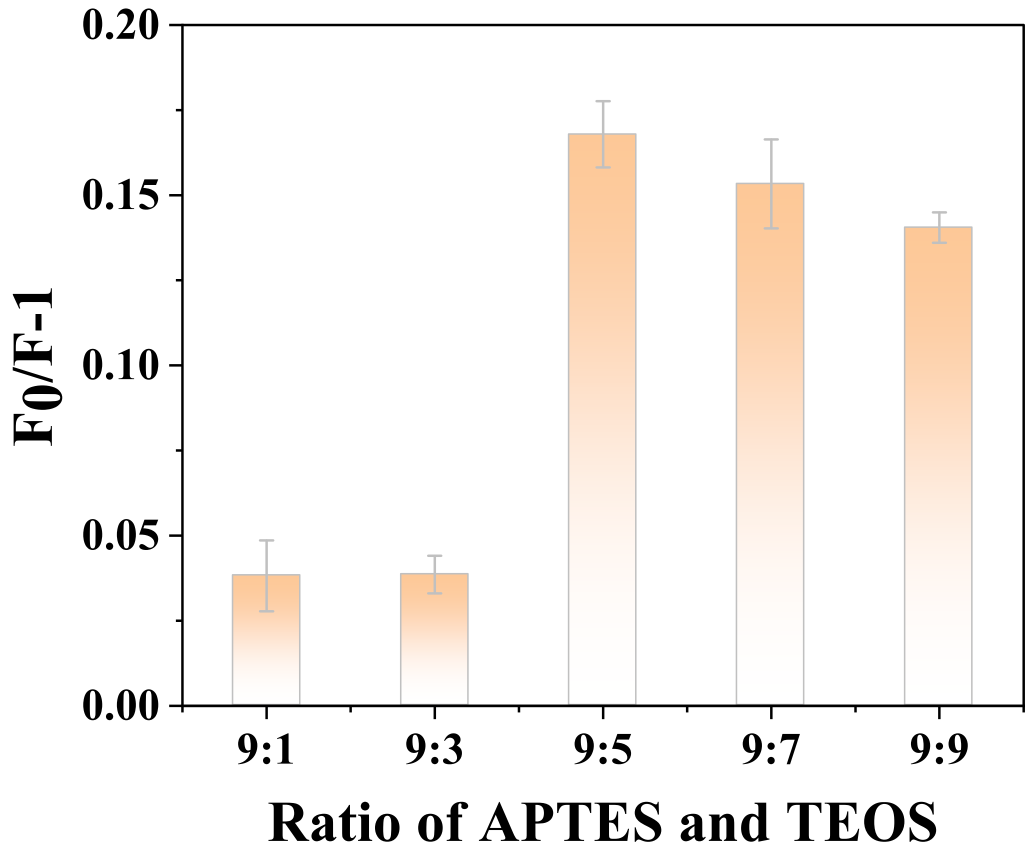

Figure S1. The effect of ratio of APTES and TEOS on the fluorescence of MIPS

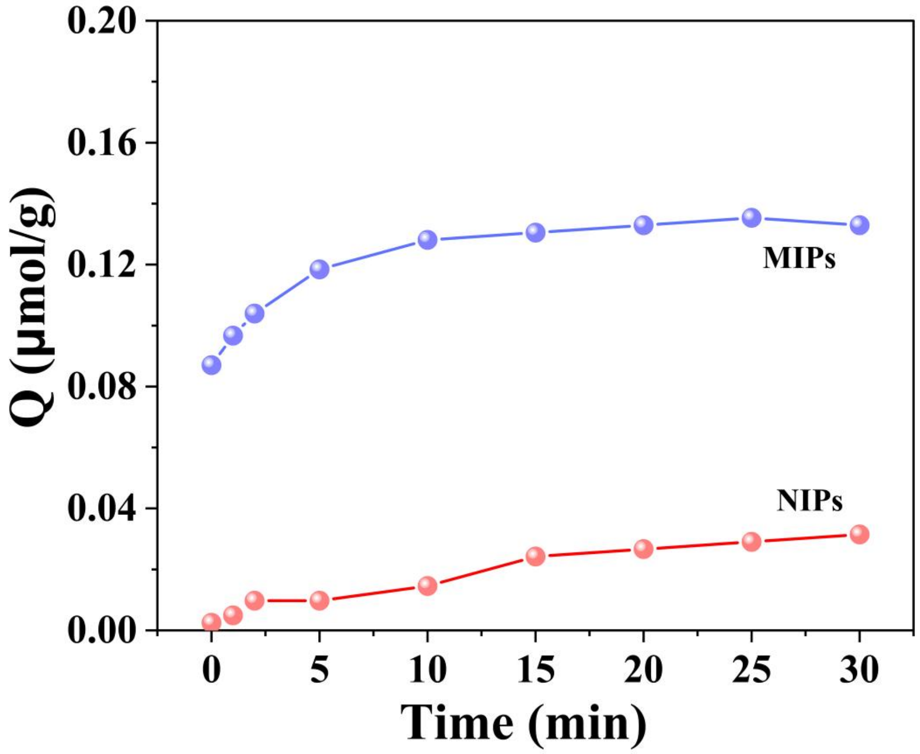

Figure S2. The adsorption capacity of MIPs and NIPs for PFOA

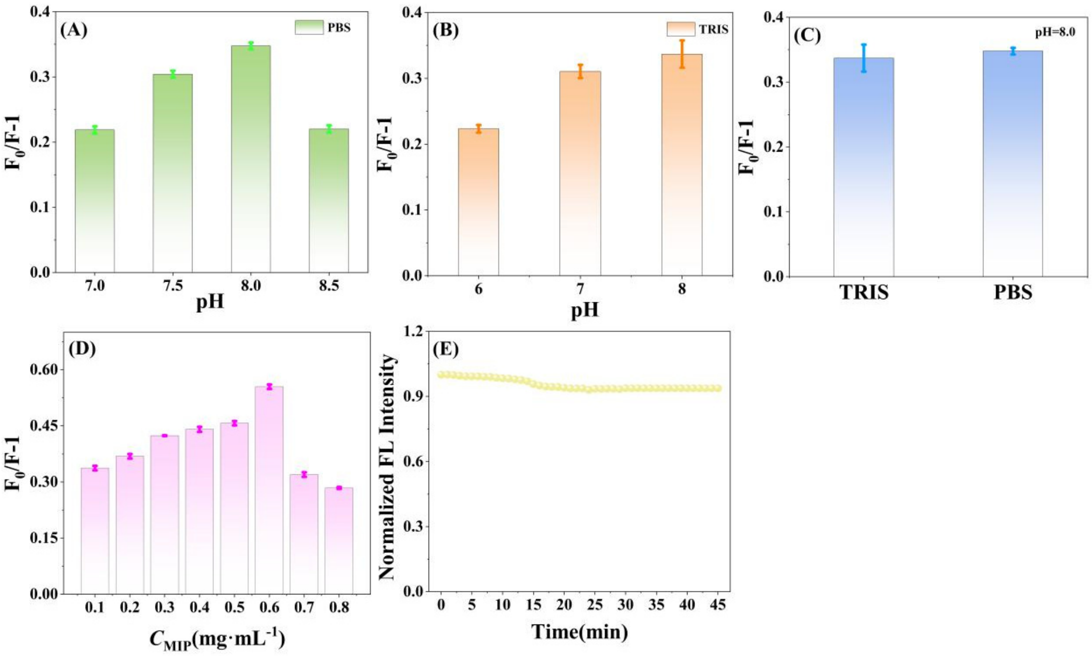

Figure S3. (A) Effect of PBS buffer at different pH values on the fluorescence intensity of the reaction system; (B) Effect of Tris buffer at different pH values on the fluorescence intensity of the reaction system; (C) Effect of Tris and PBS buffers on the fluorescence intensity of the reaction system at pH 8.0; (D) Variation of the fluorescence intensity of the reaction system with different MIP dosages; (E) Influence of equilibrium time on the fluorescence intensity of the reaction system.
